# Digitale Gesundheitsanwendungen: gesetzliche Einführung patientenzentrierter digitaler Innovationen in die Gesundheitsversorgung

**DOI:** 10.1007/s00103-021-03407-9

**Published:** 2021-09-16

**Authors:** Gottfried Ludewig, Christian Klose, Lars Hunze, Sophia Matenaar

**Affiliations:** Bundesministerium für Gesundheit, 11055 Berlin, Deutschland

**Keywords:** Digitale Gesundheitsanwendungen, DiGA, DVG, DiGAV, Fast-Track, DVPMG, Digital health applications, DiGA, Health apps, Fast track, DVG, DVPMG

## Abstract

Mit dem Digitale-Versorgung-Gesetz (DVG) und der Digitale-Gesundheitsanwendungen-Verordnung (DiGAV) wurden digitale Gesundheitsanwendungen (DiGA) definiert und als Regelleistung der gesetzlichen Krankenversicherung (GKV) eingeführt; mit dem Digitale Versorgung und Pflege – Modernisierungs-Gesetz (DVPMG) wurde der neue Leistungsbereich weiter ausgestaltet. Anwendungen, die ein zügiges dreimonatiges Prüfverfahren („Fast-Track-Verfahren“) beim Bundesinstitut für Arzneimittel und Medizinprodukte (BfArM) erfolgreich durchlaufen haben und im DiGA-Verzeichnis des BfArM gelistet sind, können nunmehr von jedem Arzt und Psychotherapeuten zulasten der GKV verordnet werden.

Auch eine Aufnahme zur Erprobung mit der Verpflichtung zur Durchführung einer Studie ist möglich. Damit hat der Gesetzgeber die dynamische Entwicklung im Bereich der mobilen Anwendungen aufgenommen und digitale Innovationen für Patienten in der Versorgung verfügbar gemacht. Der gesetzte Rahmen ist dabei so konzipiert, dass DiGA potenziell nicht nur die Therapieanteile beim Patienten unterstützen und Bereiche wie Selbstmanagement, Gesundheitskompetenz und Adhärenz stärken, sondern auch die Versorgungsabläufe zwischen Patienten und Leistungserbringern in vielfacher Weise besser gestalten können.

Die umfangreichen Vorgaben zur Interoperabilität mit Hilfsmitteln und Implantaten einerseits und der elektronischen Patientenakte (ePA) andererseits unterstützen diese Entwicklungsperspektive. Insgesamt wird es darauf ankommen, DiGA als Bestandteil nutzerfreundlicher, digital gestützter Prozesse in die Versorgung einzubinden. Dazu wird in den kommenden Jahren das initiale Regelungswerk vor dem Hintergrund der mit den neuen Produkten und dem neuen Prüfverfahren gesammelten Erfahrungen stetig angepasst und weiterentwickelt werden.

## Abstract (en)

The Digital Healthcare Act (DVG) and the Digital Health Applications Ordinance (DiGAV) defined digital health applications (DiGA) and included these as an entitlement of statutory health insurance (GKV). Further development of this new area of healthcare occurred within the Digital Healthcare and Care Modernization Act (DVPMG). Every doctor and psychotherapist can prescribe digital applications at the expense of statutory health insurance funding, provided the applications have successfully undergone a three-month test procedure at the Federal Institute for Drugs and Medical Devices (BfArM) and are listed in their DiGA directory. Provisional admissions of DiGA accompanied by scientific studies and evaluations are also possible.

The legislator has thus actively incorporated the highly dynamic technological and social development of digital innovations into healthcare by introducing mobile applications for therapeutic purposes. The legal framework is designed in such a way that DiGA can not only potentially support aspects of patient-centred care, such as strengthening of self-management, health literacy and adherence but also optimize treatment procedures and interaction between patients and service providers in many ways.

Extensive specifications for technical interoperability between DiGA and other types of medical devices and implants, as well as the electronic patient record (ePA), will accelerate the integration of DiGA into day-to-day care. Overall, it is of utmost importance to incorporate DiGA into existing care as enriching, user-friendly and digitally supported processes. Hence, the initial regulatory framework will continuously be adapted and developed in the years to come – shaped by the experiences gained with innovative products and the administrative procedures used to admit them.

## Digitale Innovation für die gesetzliche Krankenversicherung (GKV)

Mit dem Digitale-Versorgung-Gesetz (DVG) und der Digitale-Gesundheitsanwendungen-Verordnung (DiGAV) wurden digitale Gesundheitsanwendungen (DiGA) definiert und als Regelleistung der gesetzlichen Krankenversicherung (GKV) eingeführt; mit dem Digitale Versorgung und Pflege – Modernisierungs-Gesetz (DVPMG) wurde der neue Leistungsbereich weiter ausgestaltet. Anwendungen, die ein zügiges dreimonatiges Prüfverfahren („Fast-Track-Verfahren“) beim Bundesinstitut für Arzneimittel und Medizinprodukte (BfArM) erfolgreich durchlaufen haben und im DiGA-Verzeichnis des BfArM gelistet sind, können nunmehr von jedem Arzt und Psychotherapeuten zulasten der GKV verordnet werden.

Das Fast-Track-Verfahren wurde mit dem Ziel konzipiert, die Dynamik, das Innovationspotenzial und die konsequente Patientenperspektive digitaler Gesundheitsanwendungen aufzunehmen und die damit verbundenen Chancen für die Weiterentwicklung der Gesundheitsversorgung insgesamt nutzbar zu machen. „Zusätzlich zur Nutzung im zweiten Gesundheitsmarkt besteht ein gesellschaftliches Interesse, Gesundheits-Apps in die krankenkassenfinanzierte Versorgung zu implementieren, da hiermit große Nutzenpotenziale wie der niedrigschwellige Zugang zu einer Reihe von Versorgungsleistungen unabhängig von Zeit und Wohnort, die Beteiligung der Patientinnen und Patienten bei der Kontrolle und Behandlung ihrer Krankheit sowie die Erleichterung der Kommunikation und Koordination zwischen Angehörigen der Gesundheitsberufe verbunden werden“ [1].

Das in den Jahren 2019 bis 2021 entwickelte Regelungswerk setzt hierzu einen ersten Rahmen. Es leistet einen Brückenschlag zwischen technologischen Entwicklungen und veränderten gesellschaftlichen Ansprüchen auf der einen Seite und etablierten Prinzipien und Strukturen in der gesetzlichen Krankenversicherung auf der anderen. Es eröffnet einen neuen Leistungsbereich, bietet Antworten auf die wichtigsten damit verbundenen Fragen und Herausforderungen und bereitet darüber hinaus weitere Entwicklungsperspektiven für die Zukunft vor. Das gilt sowohl für den Einsatz von digitalen Anwendungen in anderen Versorgungsbereichen wie etwa der medizinischen Rehabilitation oder der Pflege als auch und besonders im Hinblick auf die Integration von DiGA und anderen Anwendungen, Diensten und Leistungen: Im Zusammenspiel von DiGA mit E-Rezept, elektronischer Patientenakte (ePA), Videosprechstunde etc. können umfassende, digitale oder digital gestützte Versorgungsabläufe entstehen.

## Ein neuer Leistungsanspruch in der GKV

**Figure 1:**
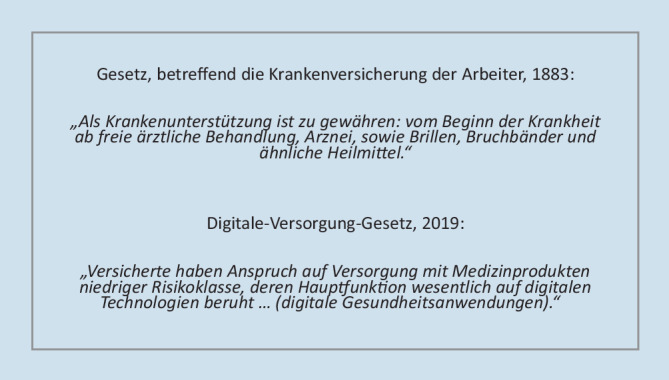

Mit dem Digitale-Versorgung-Gesetz (DVG), das am 19.12.2019 in Kraft trat, wurde ein neuer Leistungsanspruch der Versicherten in der Regelversorgung der GKV geschaffen. Erstmals seit Einführung der gesetzlichen Krankenversicherung im Jahr 1883 bezieht sich dieser Anspruch nicht auf analoge Leistungen, sondern auf digitale Produkte (Abb. 1). Versicherte haben nach dem neu eingeführten § 33a Fünftes Buch Sozialgesetzbuch (SGB V) Anspruch auf Versorgung mit DiGA: digitale Medizinprodukte, die von den Patienten oder von Leistungserbringern und Patienten gemeinsam genutzt werden. Das DVG definiert DiGA als „Medizinprodukte[n] niedriger Risikoklasse, deren Hauptfunktion wesentlich auf digitalen Technologien beruht und die dazu bestimmt sind, bei den Versicherten oder in der Versorgung durch Leistungserbringer die Erkennung, Überwachung, Behandlung oder Linderung von Krankheiten oder die Erkennung, Behandlung, Linderung oder Kompensierung von Verletzungen oder Behinderungen zu unterstützen“ (§ 33a Abs. 1 SGB V). DiGA können demnach auch Hardware wie Sensoren, Messgeräte, Übungsgeräte o. Ä. mit umfassen, wenn diese dem digitalen Produkt nachgeordnet ist, aber notwendige Daten liefert. DiGA können Medizinprodukte der Risikoklasse I oder IIa nach den jeweils geltenden Bestimmungen des europäischen Medizinprodukterechts sein.

Im DVG wurde insbesondere der Anspruch der Versicherten auf Versorgung mit DiGA geregelt, die Vergütung ärztlicher und psychotherapeutischer Leistungen im Zusammenhang mit DiGA, die Preisbildung für DiGA und das Aufnahmeverfahren in ein Verzeichnis erstattungsfähiger DiGA beim BfArM (sogenanntes Fast-Track-Verfahren). DiGA, die in diesem Verzeichnis gelistet sind, können durch Ärzte und Psychotherapeuten zulasten der GKV verordnet oder von den Krankenkassen auf Antrag genehmigt werden. Als Voraussetzung für eine Listung im DiGA-Verzeichnis werden Sicherheit und Funktionstauglichkeit gefordert, Datenschutz und Informationssicherheit, Interoperabilität und weitere Qualitätsaspekte sowie positive Versorgungseffekte. Letztere können entweder ein medizinischer Nutzen oder Struktur- und Verfahrensverbesserungen in der Versorgung sein.

Die Digitale-Gesundheitsanwendungen-Verordnung (DiGAV), in Kraft seit 21.04.2020, füllt den im DVG gesetzten Rahmen weiter aus, regelt insbesondere das Antragsverfahren, die Anforderungen an Nachweise, die Hersteller vorlegen müssen, sowie Aufbau und Inhalte des DiGA-Verzeichnisses beim BfArM.

Mit dem Digitale Versorgung und Pflege – Modernisierungs-Gesetz (DVPMG), das am 09.06.2021 in Kraft getreten ist, sind die Regelungen zu DiGA insbesondere in den Bereichen Vergütung von Leistungserbringern, Datenschutz, Informationssicherheit und Interoperabilität ergänzt und weiterentwickelt worden. Von Bedeutung ist insbesondere der Anschluss von DiGA an die elektronische Patientenakte (ePA).

Die Änderungsverordnung zur DiGAV, die im Herbst 2021 in Kraft tritt, umfasst weitere Folgeänderungen in den Bereichen Datenschutz, Informationssicherheit und Interoperabilität; sie trägt zugleich den ersten Erfahrungen des BfArM im Rahmen des Prüfverfahrens Rechnung.

## Antragsverfahren und Anforderungen an DiGA

DiGA-Hersteller können beim BfArM entweder einen Antrag auf Erprobung oder einen Antrag auf endgültige Aufnahme in das DiGA-Verzeichnis stellen. Die Anforderungen, die an die Produkte gestellt werden, sind an international anerkannten Kriterienkatalogen orientiert, im Rahmen derer in der Regel „drei übergeordnete Schwerpunktbereiche adressiert werden. Hierzu gehören die Evidenzbasierung, Vertrauenswürdigkeit und Nutzerperspektive. Bei der Bewertung der Evidenzbasierung geht es um Fragen wie die wissenschaftliche Qualität der App-Informationen (z. B. Einhaltung klinischer Leitlinien, Übereinstimmung mit den Erkenntnissen systematischer Reviews und/oder Metaanalysen, Einbindung von Angehörigen der Gesundheitsberufe und/oder medizinischen Fachgesellschaften in die Entwicklung) und den klinischen Nutzen der App (z. B. Studien zur Wirksamkeit der App). Bei der Vertrauenswürdigkeit geht es wiederum um Fragen der Funktionalität (z. B. Zuverlässigkeit der Algorithmen, Datenschutz und Datensicherheit) und Verantwortlichkeit (z. B. regulatorische Zertifizierung, Aktualität der Informationen, Transparenz). Die Nutzerperspektive behandelt dagegen Fragen der Gebrauchstauglichkeit (z. B. Benutzerfreundlichkeit, Individualisierung, Fehlerraten), des Designs und der Ästhetik sowie der wahrgenommenen Qualität der Inhalte und Funktionen“ [1].

Im Antrag werden umfassende Angaben zur DiGA, zu den Versorgungszielen, zur vorgesehenen Rolle der Leistungserbringer, zur Kompatibilität mit Betriebssystemen und Geräten etc. gemacht und damit zugleich die spätere umfassende und transparente Beschreibung der DiGA im Verzeichnis vorbereitet. Die Checklisten im Anhang der DiGAV zu den Themen Datenschutz, Informationssicherheit, Interoperabilität und Qualität werden bestätigt bzw. zukünftig z. T. Zertifikate vorgelegt. Als Nachweise für positive Versorgungseffekte werden Studien eingereicht oder alternativ, im Falle eines Antrags auf Erprobung, systematische Datenauswertungen und ein Evaluationskonzept. Das BfArM entscheidet über die Aufnahme ins Verzeichnis innerhalb von 3 Monaten nach Vorliegen der vollständigen Antragsunterlagen.

Die Datenverarbeitung durch digitale Gesundheitsanwendungen erfolgt grundsätzlich nach Maßgabe der DSGVO. Der Hersteller muss in seiner Organisation, in seinen Prozessen und in seinen Produkten die Forderungen der DSGVO umsetzen. Darüber hinaus werden in der DiGAV einige weitergehende Einschränkungen vorgenommen: Die Datenverarbeitung ist geografisch beschränkt, es darf keine Werbung geben und es sind nur bestimmte versorgungsrelevante Zwecke der Datenverarbeitung zugelassen. Datenverarbeitung darf nur stattfinden, wenn sie für den bestimmungsgemäßen Gebrauch, die Weiterentwicklung der Anwendung oder für den Einsatz der DiGA in der Versorgung benötigt wird, zum Beispiel für die Durchführung von Studien zum Nachweis positiver Versorgungseffekte oder als Grundlage für Preisverhandlungen und Abrechnung. Mit dem DVPMG wurde festgelegt, dass die konkreten Anforderungen, die zur Umsetzung der Datenschutzvorgaben an DiGA gestellt werden, künftig vom Bundesbeauftragten für den Datenschutz und die Informationsfreiheit (BfDI), BfArM und vom Bundesamt für Sicherheit in der Informationstechnik (BSI) festgelegt werden. Auf dieser Basis können von nach Art. 42 DSGVO und § 39 Bundesdatenschutzgesetz (BDSG) zugelassenen Stellen Zertifikate angeboten werden. Das BfArM wird im Antragsverfahren ab dem 01.04.2023 die Vorlage entsprechender Zertifikate verlangen. Neben den gesetzlichen Vorgaben zur Fortschreibung des datenschutzrechtlichen Rechtsrahmens sind Hersteller und BfArM auch durch externe Entwicklungen wie die Rechtsprechung des Europäischen Gerichtshofes (EuGH) aufgefordert, die Erfüllung der Anforderungen an den Datenschutz zu prüfen und ggf. nachzusteuern. Besonders deutlich wird dies etwa am Beispiel der Entscheidung des EuGH in der Rechtssache „Schrems-II“ im Hinblick auf die Nutzung von Cloud-Dienstanbietern durch Hersteller und den infolge der Entscheidung zu treffenden technischen und organisatorischen Maßnahmen zur Gewährleistung einer rechtskonformen Datenverarbeitung.

Der Hersteller muss in seiner Organisation, in seinen Prozessen und in seinen Produkten zudem die Informationssicherheit nach dem Stand der Technik umsetzen. Neben allgemeinen Vorgaben des BSI für die Entwicklung, den Betrieb und die Nutzung von Apps und Webanwendungen wurden Schwerpunkte bei den Themen Härtung (Erhöhung der Systemsicherheit) und Bibliotheken von Drittherstellern gesetzt. DiGA-Hersteller sind verpflichtet, ein Verzeichnis der genutzten Fremdsoftware zu führen und auf Verlangen vorzulegen. Die Durchführung von Penetrationstests (Prüfung der Möglichkeit unautorisierten Eindringens ins System) sowie (ab 2022) ein Informationssicherheitsmanagementsystem sind verpflichtend. Mit dem DVPMG wurde festgelegt, dass die konkreten Anforderungen, die zur Gewährleistung der Informationssicherheit an DiGA gestellt werden, künftig von BSI, BfArM und BfDI festgelegt werden. Das BSI wird hierzu Prüfprozesse und ein Zertifikat anbieten. Ab 01.01.2023 wird das BfArM im Antragsverfahren die Vorlage eines entsprechenden Zertifikates verlangen.

**Figure 2:**
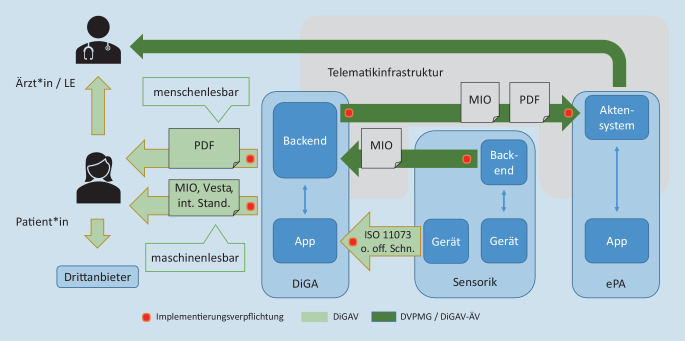

Im Bereich Interoperabilität setzt die DiGAV auf Offenheit der Anwendungen, d. h. auf die Umsetzung bzw. Förderung der Entwicklung von Standards (Abb. 2). DiGA müssen für die Aufnahme in das DiGA-Verzeichnis nachweisen, dass sie in Bezug auf 3 ausgewählte Fragestellungen interoperabel gestaltet sind:

- Die DiGA erlaubt es dem Versicherten, therapierelevante Auszüge der über die DiGA erhobenen Daten in menschenlesbarer und ausdruckbarer Form aus der DiGA auszuspielen, sodass er diese zu eigenen Zwecken nutzen oder an einen Arzt oder andere Dritte weitergeben kann.
- Die DiGA erlaubt es dem Versicherten, die über die DiGA erhobenen Daten in einem maschinenlesbaren, interoperablen Format (Syntax und Semantik) aus der DiGA auszuspielen, sodass der Versicherte oder ein vom Versicherten berechtigter Dritter diese Daten in anderen digitalen Produkten weiterverarbeiten kann.
- Wenn die DiGA über eine Frontendschnittstelle Daten aus vom Versicherten genutzten Medizingeräten oder vom Versicherten getragenen Sensoren zur Messung und Übertragung von Vitalwerten (Wearables) bezieht, kann sie diese Geräte (auch) über eine interoperable Schnittstelle ansprechen.

Mit der maschinenlesbaren Exportschnittstelle, das wurde mit dem DVPMG geregelt, werden DiGA ab 01.01.2023 auch an die ePA angebunden. Die Umsetzung der interoperablen Exportschnittstelle erfolgt als medizinische Informationsobjekte nach § 355 SGB V Abs. 2a.

Zukünftig sollen Versicherte die Möglichkeit erhalten, nicht nur Daten aus DiGA in ihre ePA zu übertragen, sondern auch Daten aus ihren Hilfsmitteln und Implantaten in DiGA zu nutzen. Der mit dem DVPMG neu eingeführte § 374a SGB V gibt dazu vor, dass ab Juli 2024 internetfähige Hilfsmittel und Implantate, die zulasten der GKV abgegeben werden, eine offene Backendschnittstelle für DiGA anbieten müssen. Wenn der Versicherte das wünscht, kann eine von ihm genutzte DiGA dort Daten abrufen, die sie zur Erzielung des positiven Versorgungseffekts benötigt.

Weitere Qualitätsanforderungen an DiGA umfassen insbesondere die Robustheit gegen Störungen und Fehlbedienungen, die Werbefreiheit, die Unterstützung der Versicherten bei der Nutzung, die bedienfreundliche Gestaltung, die Unterstützung der Leistungserbringer, Maßnahmen zur Patientensicherheit und die Fundierung der Inhalte und Funktionen der DiGA auf aktuellem qualitätsgesicherten medizinischen Wissen.

## Nachweis positiver Versorgungseffekte

**Figure 3:**
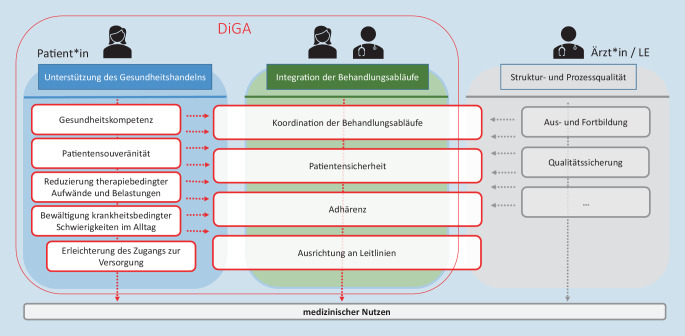

Nach § 139e Abs. 2 SGB V müssen DiGA für die Aufnahme in das DiGA-Verzeichnis positive Versorgungseffekte nachweisen, welche als „entweder ein medizinischer Nutzen oder eine patientenrelevante Struktur- und Verfahrensverbesserung in der Versorgung“ definiert werden (Abb. 3). Letztere werden in der DiGAV § 3 Abs. 8 genauer ausgestaltet: „Die patientenrelevanten Verfahrens- und Strukturverbesserungen in der Versorgung nach Absatz 1 sind im Rahmen der Erkennung, Überwachung, Behandlung oder Linderung von Krankheiten oder der Erkennung, Behandlung, Linderung oder Kompensierung von Verletzungen auf eine Unterstützung des Gesundheitshandelns der Patienten oder eine Integration der Abläufe zwischen Patientinnen und Patienten und Leistungserbringern ausgerichtet und umfassen insbesondere die Bereiche der:

1. Koordination der Behandlungsabläufe
2. Ausrichtung der Behandlung an Leitlinien und anerkannten Standards
3. Adhärenz
4. Erleichterung des Zugangs zur Versorgung
5. Patientensicherheit
6. Gesundheitskompetenz
7. Patientensouveränität
8. Bewältigung krankheitsbedingter Schwierigkeiten im Alltag
9. Reduzierung der therapiebedingten Aufwände und Belastungen der Patienten und ihrer Angehörigen.“

Der Hersteller kann frei wählen und entweder den Nachweis eines medizinischen Nutzens erbringen oder den Nachweis, dass patientenrelevante Struktur- und Verfahrensverbesserungen erzielt werden. Der positive Versorgungseffekt, der gezeigt werden soll, muss mit der Zweckbestimmung des Medizinprodukts konsistent sein und für eine nach ICD-10 definierte Patientengruppe geführt werden. Vorzulegen ist eine vergleichende Studie, die für die Patientengruppe eine Überlegenheit gegenüber Nichtanwendung der DiGA zeigt. Die Nichtanwendung kann entweder eine Nichtbehandlung oder eine Behandlung ohne DiGA oder eine Behandlung mit einer anderen endgültig gelisteten DiGA sein. Die gewählte Vergleichsgruppe soll an der Versorgungsrealität orientiert sein. Bei DiGA, die diagnostische Instrumente darstellen oder enthalten, wird zusätzlich die Ermittlung der Sensitivität/Spezifität durch eine entsprechende Studie verlangt. DiGA können auch für bis zu 12 bzw. bei einer Verlängerung maximal bis zu 24 Monate zur Erprobung in das Verzeichnis aufgenommen werden. In diesem Fall legt der Hersteller systematische Datenauswertungen zur Nutzung der DiGA und eine entsprechende Studienplanung vor und reicht die Studienergebnisse zum Ende der Erprobungszeit zur Prüfung ein. Die Durchführung der Studien ist auf Deutschland beschränkt bzw. auf Länder, für die der Nachweis einer Übertragbarkeit auf den deutschen Versorgungskontext erbracht werden kann. Die Studien werden in einem anerkannten Studienregister registriert und die Ergebnisse veröffentlicht.

## Preisbildung und Verordnung

Die Preisbildung für DiGA folgt den Maßstäben, die in einer Rahmenvereinbarung zwischen GKV-Spitzenverband (GKV-SV) und Herstellern festgelegt werden. Im ersten Jahr der Erstattungsfähigkeit setzt der Hersteller auf dieser Grundlage den Preis für sein Produkt fest, sofern die zwischen GKV-SV und Herstellerverbänden zu schließende Rahmenvereinbarung keine gruppenbezogenen Höchstpreise vorsieht. Ab dem 13. Monat der Erstattungsfähigkeit gelten die zwischen GKV-SV und Hersteller verhandelten Preise. In die Preisbildung einfließen können beispielsweise die von der DiGA bisher nachgewiesenen positiven Versorgungseffekte, die auf dem freien Markt geltenden Preise, die Preise in anderen Ländern oder sonstige in der Rahmenvereinbarung beschriebene Anforderungen. Hersteller und GKV-SV sollen zudem erfolgsabhängige Preisbestandteile vereinbaren. Mit dem Konzept der Rahmenvereinbarung wird die Aufgabe des Interessenausgleichs zwischen Innovationsoffenheit und Kosteneffizienz dabei in die Hände der Selbstverwaltung gelegt.

Die ärztliche Verordnung von DiGA ist zunächst übergangsweise auf Papier unter Nutzung des „Muster 16“ umgesetzt worden; der weitere Einlösungs- und Abrechnungsweg wird im Rahmen eines Modellprojekts von Kassen und Herstellern per Rezeptscan über die Kassen-Apps und Ausgabe eines Freischaltungscodes durch die Kassen angeboten. Das DVPMG gibt vor, dass DiGA ab dem 01.01.2023 per E‑Rezept verordnet werden. Die gematik GmbH wird die dafür erforderlichen Spezifikationen zum 01.01.2022 veröffentlichen.

## Innovative Produkte zügig ins System bringen

**Figure 4:**
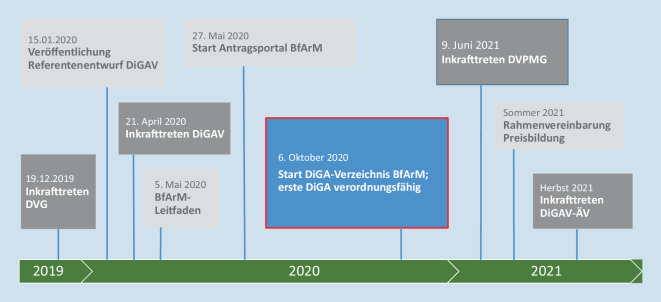

Nach Inkrafttreten der DiGAV am 21.04.2020 wurde das weitere Fast-Track-Verfahren zügig aufgebaut (Abb. 4). Der Leitfaden des BfArM mit umfassender Erläuterung des Verfahrens und der zu erfüllenden Anforderungen wurde am 05.05.2020 veröffentlicht, das Antragsportal des BfArM ging am 27.05.2020 online, das DIGA-Verzeichnis mit den 2 ersten verordnungsfähigen DiGA am 06.10.2020. Antragsportal und Leitfaden liegen auch in englischer Sprache vor. Mit dem DiGA-Verzeichnis werden umfassende und allgemeinverständliche Informationen zu jeder gelisteten DiGA öffentlich zur Verfügung gestellt. Zusätzlich wird die Weiterverbreitung der Informationen durch eine Schnittstelle für öffentliche und gemeinnützige Institutionen unterstützt.

Das beim BfArM errichtete Verfahren wurde konzipiert, um die hoch dynamische Entwicklung im Bereich der mobilen Anwendungen aufzunehmen, einen Anreiz für die Entwicklung besonders guter und sicherer Angebote zu schaffen und diese innovativen digitalen Medizinprodukte in einem transparenten, zügigen Verfahren in die Versorgung zu bringen. Mit der Formulierung umfassender Qualitäts- und Transparenzanforderungen an die Produkte und an die Prozesse beim Hersteller wurden die notwendigen Voraussetzungen für einen Einsatz in der Gesundheitsversorgung und für die notwendige Akzeptanz bei Patienten und Leistungserbringern geschaffen. Auf dieser Basis aufsetzend eröffnet die Erprobungsregelung die Möglichkeit einer vorläufigen Aufnahme in das Verzeichnis und die Durchführung einer bei Antragstellung erst im Planungsstadium befindlichen Studie. Junge Unternehmen mit innovativen Produkten, die sich für den Weg in die GKV entscheiden, können so in frühem Stadium an die Anforderungen und Rahmenbedingungen im System herangeführt werden und in den notwendigen gemeinsamen Lern- und Entwicklungsprozess mit Patienten, Leistungserbringern und Kostenträgern eintreten. Ende Mai 2021, ein Jahr nach Start des Verfahrens beim BfArM, deutet die Bilanz von insgesamt 72 eingereichten, davon bisher 30 zurückgezogenen, 3 abgelehnten und 15 positiv beschiedenen Anträgen darauf hin, dass der gesetzte Rahmen in diesem Sinne funktioniert und in seiner mehrfachen Funktion als Einladung, Hilfestellung und zugleich nicht unerhebliche Herausforderung für die neu in den ersten Gesundheitsmarkt eintretenden Unternehmen seine Wirkung zeigt.

## Perspektive der Patienten

Mit dem neu in das SGB V eingeführten Begriff der „positiven Versorgungseffekte“, welcher ein medizinischer Nutzen oder auch, diesem gleichwertig an die Seite gestellt, eine „patientenrelevante Struktur- und Verfahrensverbesserung“ sein kann, sind zudem erstmals die Prozesse und Therapieanteile beim Patienten systematisch in den Fokus gerückt und zu einer Grundlage für die Beurteilung der Erstattungsfähigkeit einer Leistung in der gesetzlichen Krankenversicherung erhoben worden (Abb. 3). Damit wurde das Primat der auf medizinische Ergebnisparameter fokussierten Nutzenbewertung durchbrochen und das Leistungsangebot der GKV geöffnet zugunsten der Bedarfe und Perspektiven der Patienten.

Die neuen digitalen Produkte stellen erweiterte Möglichkeiten bereit, die Kommunikation zwischen Patienten und Leistungserbringern zu verbessern, Laienverständlichkeit und Praxisbezug zu verwirklichen und zwischen der medizinischen Fachwelt auf der einen Seite und dem Alltagsverständnis der Patienten auf der anderen Seite zu vermitteln. DiGA können die Nutzer im Alltag mit einfachen, verständlichen Informationen, Hinweisen, Erinnerungen, Fragen unterstützen, evidenzbasierte, leitlinienbasierte Medizin und qualitätsgesicherte Gesundheitsinformationen in leicht zugängliche, kontextsensitive Form bringen, Anleitungen und Erklärungen bereitstellen, die den Patienten in seinem alltäglichen Umgang mit der Erkrankung unterstützen, dabei Gesundheitswissen anlassbezogen und individuell aufbauen, zu Verhaltensänderungen motivieren und sie einüben. Digitale Gesundheitsanwendungen haben damit das Potenzial, substanzielle Beiträge zur Verbesserung der Adhärenz, zur Stärkung der Gesundheitskompetenz und Patientensouveränität, zur Förderung einer leitliniengerechten und gut abgestimmten Behandlung zu leisten.

## Nukleus für digitale Versorgungsprozesse

**Figure 5:**
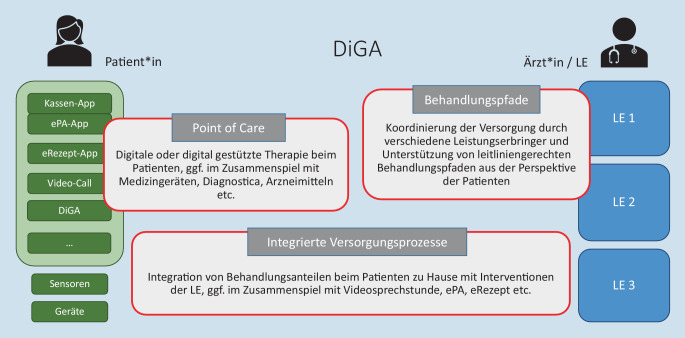

DiGA sind darüber hinaus in der Lage, vielfältige und auch umfassende Versorgungskonzepte abzubilden (Abb. 5). Die bisher in das Verzeichnis aufgenommenen DiGA fokussieren überwiegend auf den Point of Care beim Patienten und setzen hier auf Therapieunterstützung (Übungen, Tagebuch, Selbstmanagement) oder bieten eigenständige Therapie im Bereich der psychischen Erkrankungen an.

Grundsätzlich jedoch sind DiGA weiter gefasst. Durch ihre Definition als digitale Medizinprodukte, die von den Patienten oder durch Patienten und Leistungserbringer gemeinsam genutzt werden, sowie durch die umfangreichen Vorgaben zur Interoperabilität, die Anbindung an die ePA und die flankierenden Vergütungsregelungen für Leistungserbringer sind sie als Nukleus für integrierte, digital gestützte, patientenzentrierte Versorgungsprozesse angelegt.

Im Versorgungprozess sind ganz unterschiedliche Schwerpunkte möglich. Eine DiGA kann auf das Selbstmanagement chronisch Erkrankter fokussieren und im Zusammenspiel mit Sensoren und Medizingeräten den Point of Care beim Patienten unterstützen; sie kann Patienten über leitliniengerechte Behandlungspfade zwischen Leistungserbringern steuern, zur engmaschigen Verlaufsüberwachung in der akuten schweren Erkrankung oder in der Nachsorge dienen, mit Langzeitmessungen oder -erhebungen ärztliche Diagnosen unterstützen, häusliche Übungen nach Rehabilitation oder im Rahmen einer Heilmittelbehandlung anleiten und motivieren, Therapieanteile bei Patient und Leistungserbringer integrieren u. v. m.

Um die umfassenden Potenziale von DiGA zur Entfaltung zu bringen, müssen die Regelungen zum Fast-Track wie auch die weiteren Rahmenbedingungen im Gesundheitssystem Schritt um Schritt weiterentwickelt werden, auf der technischen und organisatorischen Ebene ebenso wie in den Vertrags- und Vergütungsstrukturen und den berufsrechtlichen Vorgaben bis hin zu den übergreifenden Fragen der Nutzung von Gesundheitsdaten für eine bessere Versorgung und Medizin. Insbesondere müssen DiGA in den kommenden Jahren schrittweise in die wachsende E‑Health-Infrastruktur integriert und technisch wie inhaltlich in ein dynamisches Zusammenspiel mit übergreifenden Versorgungprozessen gebracht werden so, dass nicht nur für die Patienten, sondern in gleichem Maße auch für die Leistungserbringer gute und bedarfsgerechte Abläufe entstehen, die den Behandlungsalltag wirksam unterstützen. Ein mögliches Zielbild: „Mein nächster Patient, Herr K., sitzt mit mir am Tisch und berichtet, dass er gut mit der gewählten Therapie zurechtkommt, er ist sehr gespannt, ob die Werte besser geworden sind. Alle Zielparameter sind im grünen Bereich. Ich gebe Herrn K. den Blick auf den Bildschirm frei und zeige ihm den sehr erfreulichen Stand der Dinge. Die grafische Darstellung ist auch für Patienten selbsterklärend. … Die Therapieerfolgsgrafik und die vervollständigte Impfübersicht werden automatisch in seine nationale Gesundheitsakte übertragen, seine Patienten-App zeigt den grünen Haken, der besagt, dass aktuell alle relevanten Ziele erreicht sind. … Noch schnell den elektronischen Arztbrief, der vom System weitgehend selbst erstellt wird, vervollständigen. Ein Klick, und alle Daten sind komplett in die nationale Gesundheitsakte übertragen, die den anderen weiterbehandelnden Ärzten sofort vollumfänglich zur Verfügung steht“ [2].

## Zukünftige Herausforderungen und Chancen

Die Integration von DiGA in größere Versorgungsabläufe wird viel Neues verlangen, von den Herstellern und Produkten wie auch von den Leistungserbringern. Im Bereich der Evidenznachweise stellen die dynamischen Innovationszyklen der Produkte bereits heute eine Herausforderung dar. Zukünftig wird sich die Frage nach geeigneten Evidenznachweisen noch verstärken: Je tiefer DiGA in die Versorgung integriert werden, je stärker sie an Therapieabläufen und Therapieentscheidungen beteiligt sind und zu einem Bestandteil komplexer Interventionen werden, desto schwieriger wird sich der Nachweis der spezifischen Wirkungen und Effekte der digitalen Produkte gestalten.

Zugleich werden jedoch auch die Möglichkeiten wachsen, mithilfe von DiGA geeignete Ergebnisparameter zu erheben und die Effekte von Versorgungskonzepten systematisch zu messen und auszuwerten. In § 134 Abs. 1 SGB V ist mit der Aufforderung an die Verhandlungspartner, auch erfolgsabhängige Preisbestandteile für DiGA zu vereinbaren, diese Dimension bereits angelegt. In Bereichen wie beispielsweise der Diabetesversorgung eröffnen sich hier neue Perspektiven. „Die DiGA bietet das Potenzial, Ergebnisindikatoren automatisiert und mit geringem administrativen Aufwand digital zu erfassen und zu dokumentieren, um sie zu Zwecken einer erfolgsabhängigen Vergütung zu analysieren … Dauerhaft stabil bleibende und nicht ausschlagende Blutzuckermesswerte als Ergebnisindikator für ein Pay-for-Performance-Modell können dabei nicht nur vergleichsweise einfach über einen längeren Zeitraum beobachtet werden, sondern zugleich neben den vom Arzt erhobenen, perspektivisch in die ePA eingetragenen und von dort in die DiGA übertragenen Werte des HbA1c als weiterer Ergebnisindikator ergänzt werden. Zudem kann die für den langfristigen Erfolg der Diabetes-Behandlungen wichtige Anpassung des Lebensstils des Patienten/der Patientin … ebenfalls automatisch erfasst und dokumentiert werden, ohne dass für den Arzt oder sonstiges Personal ein zusätzlicher Aufwand entsteht. Gleichzeitig kann eine Vielzahl an Indikatoren mit Hilfe von Patient Reported Outcomes unter Anwendung von Fragebögen, z. B. zur gesundheitsbezogenen Lebensqualität, gemessen werden. Diese können zu festgelegten Zeitpunkten vergleichsweise einfach in der DiGA digital abgefragt und dokumentiert werden, sodass auch hier kaum administrativer Aufwand entsteht“ [3].

## Gesetzgebung als agiler Prozess

Das bisher gesetzte Regelwerk ist nicht als abschließend zu verstehen, sondern im Gegenteil als Ausgangspunkt eines kontinuierlichen Prozesses der Beobachtung, Nachjustierung, Korrektur und Erweiterung, der die dynamischen Veränderungen in Technologie und Gesellschaft, die Fortschritte in der Medizin wie auch die Erwartungen und Erfahrungen im System immer von Neuem aufnimmt und reflektiert. Diese Vorgehensweise wird auch von den Akteuren im System weitgehend positiv reflektiert und begleitet. „Der GKV-Spitzenverband begrüßt die gesetzgeberischen Aktivitäten, mit denen die Digitalisierung des Gesundheitswesens und der Pflege vorangetrieben werden soll. Positiv ist, dass dies in einem iterativen Prozess erfolgt. Das bietet Chancen, Erfahrungen zu sammeln, aktuelle Entwicklungen aufzunehmen und Fehlentwicklungen frühzeitig zu korrigieren“ [4].

Die ersten entscheidenden Schritte sind getan und haben die gesetzliche Krankenversicherung erfolgreich für digitale Innovation in der Hand der Patienten und für die damit verbundenen Chancen für eine bessere Gesundheitsversorgung geöffnet. „Fasst man die durch das DVG bewirkten Neuerungen zusammen, ist festzustellen, dass die Neuregulierung sowohl mit Blick auf die digitale Innovationsförderung von Gesundheitsapps als auch im Hinblick auf die generelle Regelungsstruktur des SGB V von erheblichem Neuerungswert ist. Dies gilt mit Blick auf das Recht der GKV in dreierlei Hinsicht: Erstens erweist sich die faktische Erweiterung des Nutzenbegriffes auf Aspekte der Gesamtversorgungsstruktur sowie Versorgungsbedingungen als wegweisend. Zweitens ist die erfolgte Verkoppelung von Anreizstrukturen für die Entwicklung und Optimierung von Gesundheitsapps mit Vorgaben zu deren Qualität und Sicherheit als gelungen anzusehen, weil hierdurch Innovationsförderung sowohl mit Blick auf die Verbesserung als auch Entwicklung von Gesundheitsapps betrieben wird. Drittens wird im Wege der Zuständigkeitszuweisung zur Führung des Verzeichnisses digitaler Gesundheitsanwendungen an das Bundesinstitut für Arzneimittel und Medizinprodukte geschickt die Problematik der umstrittenen Legitimation der gemeinsamen Selbstverwaltung im Krankenversicherungsrecht umschifft, was ebenfalls Vorbildcharakter für andere Bereiche des Krankenversicherungsrechts haben könnte“ [5].
